# Childhood Trauma, Emotion Regulation, and Pain in Individuals With Alcohol Use Disorder

**DOI:** 10.3389/fpsyt.2020.554150

**Published:** 2020-10-30

**Authors:** Justyna Zaorska, Maciej Kopera, Elisa M. Trucco, Hubert Suszek, Paweł Kobyliński, Andrzej Jakubczyk

**Affiliations:** ^1^Department of Psychiatry, Medical University of Warsaw, Warsaw, Poland; ^2^Department of Psychology and the Center for Children and Families, Florida International University, Miami, FL, United States; ^3^Department of Psychiatry, Addiction Center, University of Michigan, Ann Arbor, MI, United States; ^4^Department of Psychology, University of Warsaw, Warsaw, Poland; ^5^Laboratory of Interactive Technologies, National Information Processing Institute, Warsaw, Poland

**Keywords:** childhood trauma, pain, emotion regulation, alcohol use disorder, mediation

## Abstract

**Introduction:** Several studies have confirmed that the experience of childhood trauma, poor emotion regulation, as well as the experience of physical pain may contribute to the development and poor treatment outcomes of alcohol use disorder (AUD). However, little is known about how the joint impact of these experiences may contribute to AUD.

**Objectives:** To analyze associations between childhood trauma, emotion regulation, and pain in individuals with AUD.

**Methods:** The study sample included 165 individuals diagnosed with AUD. The Childhood Trauma Questionnaire (CTQ) was used to investigate different types of trauma during childhood (physical, emotional, and sexual abuse and neglect), the Brief Symptom Inventory was used to assess anxiety symptoms, the Difficulties in Emotion Regulation Scale (DERS) was used to assess emotional dysregulation, and the Pain Resilience Scale and Pain Sensitivity Questionnaire were used to measure self-reported pain tolerance and sensitivity.

**Results:** Childhood emotional abuse (CTQ subscale score) was positively associated with anxiety, anxiety was positively associated with emotional dysregulation, and emotional dysregulation was negatively associated with pain tolerance. Accordingly, there was support for a significant indirect negative association between childhood emotional abuse and pain tolerance. The serial mediation statistical procedure demonstrated that anxiety and emotional dysregulation mediated the effect of childhood emotional abuse on pain resilience among individuals with AUD.

**Conclusions:** Targeting emotional dysregulation and physical pain that can result from childhood trauma may have particular therapeutic utility among individuals treated for AUD.

## Introduction

According to the World Health Organization (WHO), child maltreatment can be defined as the “physical and emotional mistreatment, sexual abuse, neglect and negligent treatment of children, as well as to their commercial or other exploitation.” (1). Child maltreatment is the most prevalent cause of childhood trauma (2, 3). In general, childhood trauma has been considered a risk factor for various negative outcomes through the life span (4). For example, it has been widely reported that childhood maltreatment increases the risk of alcohol use disorder (AUD) (5–9). In addition, individuals with AUD are more likely to report childhood maltreatment compared to healthy controls (10–13), with one study finding that more than 50% of a sample of individuals with AUD endorsed childhood trauma (10). Prior work supports links between childhood trauma and (1) early initiation of alcohol use (14), (2) earlier age at AUD onset, (3) longer duration and more severe AUD symptoms (11, 15, 16), and (4) poor AUD treatment outcomes (i.e., higher risk of relapse after treatment) (10, 17).

Another factor relevant to the development, course, and treatment outcomes of AUD is emotion dysregulation (18–21). Emotion regulation involves awareness, understanding and acceptance of emotions, ability to control behaviors when experiencing emotional distress, and capacity to use adaptive strategies to modulate emotional responses (22). Difficulties in emotion regulation were consistently recognized as a risk factor of developing AUD symptoms (18, 19). Moreover, prior work consistently indicates that childhood trauma leads to early emotion regulation difficulties or to emotion dysregulation in later life (23). Individuals with severe childhood abuse or neglect report more maladaptive emotion regulation strategies in adulthood (24–26) compared to those without a history of childhood maltreatment. A recent review by Janiri et al. (27) concludes that childhood trauma is closely linked to emotional hyperreactivity and affective lability. Furthermore, exposure to traumatic life events in childhood is associated with symptoms of mixed mood states, and this effect is mediated by emotional hyperreactivity. Importantly, among different types of childhood trauma, emotional abuse was most strongly associated with negative affectivity and emotion dysregulation in later life (28–30). Finally, prior work supports the role of emotion dysregulation as a possible mechanism by which early emotional and physical maltreatment impacts later substance use disorder (31, 32).

Several neurobiological studies support complementary underlying mechanisms that may contribute to the links found between childhood trauma and emotion dysregulation. For example, neuroimaging studies conducted on individuals with a history of childhood trauma revealed alterations in brain regions responsible for emotion processing [i.e., decreased hippocampal and amygdalar volumes (33)], as well as decreased gray matter volume of the left dorsolateral pre-frontal cortex, regardless of depressive symptoms (34). Functional neuroimaging investigations conducted on patients with post-traumatic stress disorder (PTSD) supported hyperactivity in the amygdala and insula in response to negative content and low engagement of the anterior cingulate cortex (ACC) during negative emotion processing (35). In another study (36), effects of childhood adversity on the volume of subregions in the hippocampus were investigated. Among healthy individuals, childhood trauma was associated with bilaterally smaller cornu ammonis (CA1), presubiculum, and subiculum subfields. Interestingly, in patients with bipolar disorder, this effect was not demonstrated. The areas of interest in this study closely parallel brain regions involved in fear and stress response (e.g., the amygdala, the medial pre-frontal cortex, the ACC) and emotion regulation.

Pain is a multifaceted experience with both sensory and affective components. Emotional factors can strongly impact pain perception. Namely, prior work indicates that negative emotional states tend to increase pain perception, while positive states tend to reduce the experience of pain (37, 38). Brain imaging studies revealed that attentional and affective states may alter activity in afferent pain pathways. For example, functional magnetic resonance imaging (fMRI) demonstrated that positive mood states decrease pain-related activity within the ACC, the medial thalamus, and primary and secondary somatosensory cortices, while negative mood states enhance pain-evoked activity in the ACC, the insular cortex (IC), frontal brain regions, and the hippocampus (39, 40). In addition, there is growing evidence that patients with chronic pain may develop anatomical changes in regions involved in cognitive and emotional modulation of pain (e.g., the dorsolateral and medial pre-frontal cortex, the ACC, and the insula) (41). Chronic pain was also associated with gray matter loss in several of the same brain regions, especially the pre-frontal cortex, as well as the insula and the ACC (42). Additionally, diffusion-weighted imaging revealed that chronic pain may lead to disruptions in white matter tracts involved in pain processing (41, 43). Prior work has also shown that physical pain is an important factor contributing to the development and course of AUD (44–47). Moreover, overlapping neural mechanisms that contribute to the co-occurrence of AUD and chronic pain (e.g., changes in pre-frontal cortex, nucleus accumbens, and amygdala activity) indicate possible links between neurobiological systems associated with reward and stress (48).

It has been suggested that physical pain may influence the use of alcohol given the analgesic effect of ethanol (49). Moreover, prior work supports an association between pain and negative affect and emotion dysregulation, both of which are risk factors for AUD (50). Witkiewitz et al. (51) demonstrated that negative affect significantly mediated the association between severe pain and drinking outcomes among patients receiving combined pharmacotherapy and behavioral interventions. Similarly, greater emotion regulation was associated with lower severity of physical pain among individuals with AUD (52). The association between alcohol use, emotion regulation, and pain can be linked to several neurobiological regions. That is, the ACC and ICs and their connections to the pre-frontal cortex have been linked to the simultaneous regulation of emotions, pain, and alcohol drinking (53).

There are also studies supporting direct associations between childhood trauma and pain. A study conducted by Sansone et al. (54) found that adult internal medicine patients reporting specific forms of childhood trauma (i.e., witnessing violence, emotional abuse, physical abuse, or sexual abuse) had significantly higher severity ratings of pain compared to patients without a history of childhood trauma. Other findings from a group of patients with fibromyalgia demonstrate that childhood neglect may predict pain intensity. This relation was mediated by cortisol change over time such that childhood emotional and physical neglect were associated with a flattened cortisol day profile, which in turn was associated with higher daily pain (55). Furthermore, Scarinci et al. (56) observed that individuals reporting a history of child abuse, in contrast to non-abused individuals, had significantly lower pain threshold levels as assessed by finger pressure stimuli. Similarly, Pieritz et al. (57) described an association between emotional childhood abuse and decreased tolerance to heat pain. However, prior work examining the association between childhood trauma and physical pain in individuals with AUD is scarce. A notable exception is a study that demonstrated that adults with AUD and a history of childhood sexual abuse experienced more severe physical pain than adults with AUD not reporting a sexual abuse history (58).

Taken together, there is strong support for mutual associations between childhood trauma, negative affect, emotion regulation, and physical pain. However, little is known about associations between these factors among individuals with AUD. Investigating these associations in an AUD sample may be particularly important given the critical role that emotion regulation, negative affect, pain, as well as childhood trauma play in the development and course of AUD. Examination of these factors will likely have clinical significance, as prior work indicates that these factors may be addressed in alcohol treatment programs (46, 59, 60). The current study was designed to assess associations between childhood trauma, emotion dysregulation, negative affect, and pain among individuals with AUD. We hypothesized that among individuals with AUD, experiencing childhood trauma would be associated with poor emotion regulation, higher negative affect, and higher severity of physical pain. Furthermore, we hypothesized that emotional dysregulation and negative affect would mediate the relationship between childhood trauma and pain severity.

## Materials and Methods

### Participants and Procedures

A sample (165 adults 18 years or older) was recruited among patients entering an 8 week, drug-free, abstinence-based, inpatient alcohol treatment program in Warsaw, Poland. Given the overrepresentation of men in Polish inpatient alcohol treatment programs, the sample was comprised primarily of White men (88.1%). The sample was characterized by the following alcohol use characteristics: average age of alcohol drinking problems onset was 25.7 ± 9.6 years of age; the average duration of the last self-reported drinking period was 69.5 ± 196.6 (min = 1; max = 1,460) days; the maximum amount of daily alcohol consumption during the last drinking period was 285.8 ± 200.3 standard units (with 1 standard unit = 10 g of 100% ethanol according to European calculator); and the average period of abstinence at the day of the assessment was 49.2 ± 45.1 days. Accordingly, the sample represents individuals with severe AUD symptoms and severe consequences of alcohol use.

Study procedures were performed during the first 2 weeks after treatment admission. Participants had to meet criteria for an AUD based on both the International Classification of Diseases and Related Health Problems 10th Revision (61) and the MINI International Neuropsychiatric Interview (62) to be eligible for the study. Exclusion criteria consisted of the following: a history of psychosis, co-occurring psychiatric disorders requiring current medication, the presence of acute alcohol withdrawal symptoms, or the presence of a clinically significant cognitive deficit [<25 on the Mini-Mental State Examination (63)].

This study was conducted in accordance with the ethical principles described in the Declaration of Helsinki in 1964 and received ethical approval from the institution where the study took place (KB/258/2016).

### Measures

#### Psychiatric Comorbidity

Comorbidity was assessed with the Polish version of the MINI International Neuropsychiatric Interview (64). The Polish version of the Brief Symptom Inventory (BSI) (65) was utilized to assess negative affect, namely, depressive and anxiety symptoms. Both have been associated with childhood trauma, emotion dysregulation, and more severe experience of pain in prior work (66, 67). Therefore, anxiety and depression were taken into consideration in statistical models.

#### Emotion Regulation

The Polish version of the Difficulties in Emotion Regulation Scale (DERS) (68, 69) was used to assess emotion dysregulation. DERS assesses six domains of emotion dysregulation: “non-acceptance of negative emotions, inability to engage in goal-directed behaviors when experiencing negative emotions, difficulties controlling impulsive behaviors when experiencing negative emotions, limited access to effective emotion regulation strategies, and lack of own emotional awareness and clarity” (69). For the purpose of the current study, a total DERS score was used (Cronbach's α = 0.93). Higher scores on the DERS indicate *worse* emotion regulation.

#### Pain

Pain tolerance was assessed with the Pain Resilience Scale (70, 71), which measures the degree to which participants report experiencing cognitive, affective, and behavioral responses when faced with intense or prolonged pain (Cronbach's α = 0.90). Higher scores indicate greater levels of pain-specific resilience.

Pain sensitivity was assessed with the Pain Sensitivity Questionnaire. This measure consists of 17 (of which 14 are analyzed) questions reflecting self-reported subjective pain sensitivity in everyday situations (Cronbach's α = 0.92) (72, 73).

#### Childhood Trauma

The Polish version of a short form of the Childhood Trauma Questionnaire (CTQ-SF) was used to assess maltreatment history. The CTQ-SF contains 28 items that ask about experiences during childhood and adolescence. Items are rated on a 5-point Likert-type scale ranging from 1 = “never true” to 5 = “very often true.” The CTQ-SF has five factors—physical abuse, sexual abuse, emotional abuse, physical neglect, and emotional neglect—that have been empirically derived (74, 75). The five factors (range of Cronbach's α = 0.65–0.90) were analyzed separately as they constitute distinct forms of childhood trauma.

### Statistical Analysis

To test our *a priori* hypothesis that current negative affect and emotional dysregulation mediate the effect between childhood trauma and current pain tolerance, Preacher and Hayes' (2008) PROCESS SPSS macro for serial mediation (model 6) with bootstrapping (5,000 resamples with replacement) was applied. The final model (consistent with *a priori* hypotheses) was derived by examining bivariate correlations that demonstrated at least a small effect size (i.e., *r* ≥ |0.10|) to reduce the number of estimated models. Non-standardized coefficients are reported for the serial mediation model. Sex and age were included as covariates.

## Results

### Bivariate Correlations

Emotional dysregulation (DERStotal) was significantly correlated with greater anxiety (BSIanx; *r* = 0.42, *p* < 0.001), lower pain tolerance (PRS; *r* = –0.34, *p* < 0.001), higher pain sensitivity (PSQ; *r* = 0.21, *p* < 0.01), and severity of childhood emotional abuse (CTQemo; *r* = 0.19, *p* < 0.02). Other types of childhood maltreatment were not significantly correlated with emotional dysregulation (Table 1). In addition, lower pain tolerance (PRS) was significantly associated with higher anxiety (BSIanx; *r* = −0.18, *p* < 0.05) as well as severity of childhood emotional abuse (CTQemo; *r* = −0.17, *p* < 0.05). Higher pain sensitivity (PSQ) was significantly associated with higher anxiety (BSIanx; *r* = 0.19, *p* < 0.05) but not significantly correlated with childhood maltreatment.

**Table 1:** Correlations between types of childhood trauma, emotional dysregulation, pain tolerance and sensitivity, and negative affect (anxiety).

		**DERStotal**	**PRS**	**PSQ**	**BSIanx**
CTQtotal	*R*	0.150	**−0.295**	0.139	**0.266**
	*p*	0.073	**0.000**	0.098	**0.001**
CTQphys	*R*	0.026	**−**0.078	0.028	**0.169**
	*P*	0.755	0.353	0.740	**0.043**
CTQemo	*R*	**0.191**	**−0.173**	0.128	**0.338**
	*p*	**0.022**	**0.038**	0.128	**0.000**
CTQeneg	*R*	0.156	**−0.260**	0.158	**0.247**
	*p*	0.063	**0.002**	0.059	**0.003**
CTQpneg	*R*	0.116	**−0.357**	0.081	**0.183**
	*p*	0.166	**0.000**	0.339	**0.028**
CTQsex	*R*	0.137	**−**0.160	0.144	0.039
	*p*	0.101	0.056	0.087	0.645

*AUD, alcohol use disorder; BSI, Brief Symptom Inventory; anx, anxiety symptoms; DERS, Difficulties in Emotion Regulation Scale; PRS, Pain Resilience Scale; PSQ, Pain Sensitivity Questionnaire; CTQ, Childhood Trauma Questionnaire; CTQemo, emotional abuse; CTQphys, physical abuse; CTQsex, sexual abuse; CTQeneg, emotional neglect; CTQpneg, physical neglect. Statistically significant correlations are indicated in bold*.

Thus, subsequent mediation models focused on severity of childhood emotional abuse as the predictor, anxiety, and emotional dysregulation as potential mediators (consistent with study hypotheses), and pain tolerance as the outcome.

### Mediation Model

**Figure 1:**
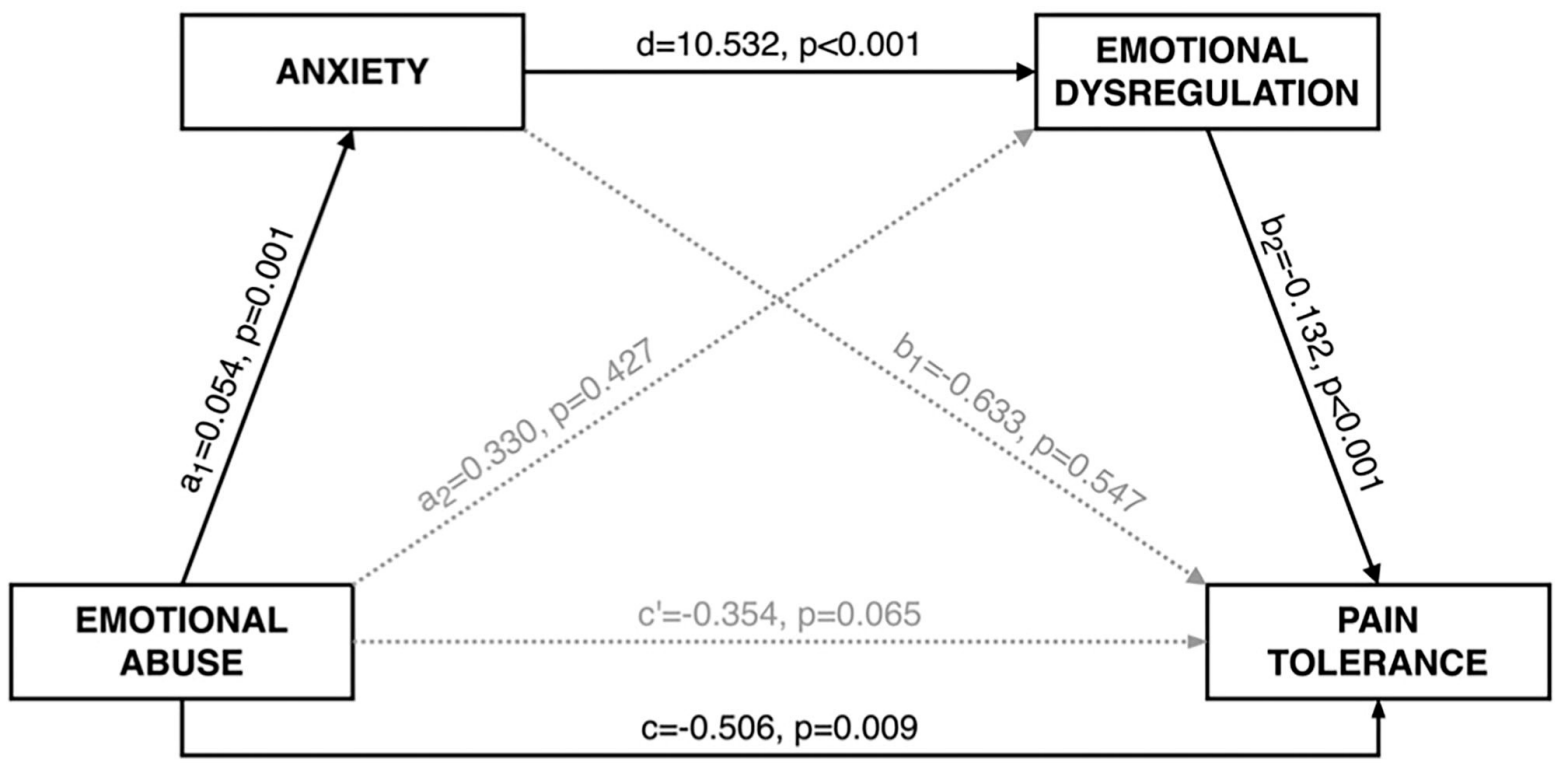
Serial mediation model. Indirect effect of childhood emotional abuse during childhood on pain tolerance *via* anxiety and emotional dysregulation at the moment of the study. Non-standardized coefficients are reported.

The severity of childhood emotional abuse, current level of anxiety, emotional dysregulation, and the covariates (sex and age) explained approximately 16% of the variance in current pain tolerance (*R*^2^ = 0.156, *F*_[5, 138]_ = 5.094, *p* < 0.001; Figure 1). Childhood emotional abuse was positively associated with anxiety, anxiety was positively associated with emotional dysregulation, and emotional dysregulation was negatively associated with pain tolerance. Moreover, the indirect effect of childhood emotional abuse on pain tolerance was significant using the bootstrapping procedure (non-standardized indirect effect was −0.075, the 95% confidence interval ranged from −0.162 to −0.021). Neither of the other two indirect effects (a_1_b_1_ and a_2_b_2_ in Figure 1) nor the direct effect of childhood emotional abuse on pain tolerance (c′ in Figure 1) was significant. Thus, there was support for serial mediation whereby the effect of childhood emotional abuse on pain tolerance operated *via* anxiety and emotional dysregulation.

## Discussion

Findings support an association between childhood emotional abuse, higher emotional dysregulation, and lower pain tolerance among adult individuals with AUD. More specifically, results show a positive association between severity of childhood emotional abuse and greater anxiety. In turn, greater anxiety was associated with emotional dysregulation, which in turn was negatively associated with pain tolerance. There was also support for the role of anxiety and emotional dysregulation as serial mediators in the association between childhood emotional abuse and pain tolerance. To the best of our knowledge, this is the first study to investigate interrelations between childhood trauma, emotional dysregulation, and physical pain among individuals with AUD. This is particularly meaningful within this clinical population given that these factors have all been demonstrated to be critical in the development and course of AUD.

Childhood maltreatment has been shown to be strongly associated with numerous negative health-related outcomes across development. For example, childhood trauma has been linked to emotion regulation difficulties and increased risk of developing AUD (76). Moreover, prior work shows that individuals who experienced maltreatment in childhood report more physical pain in adulthood in comparison to individuals without a maltreatment history (54, 66, 77). Similarly, patients with chronic pain are more likely to report a history of childhood abuse or neglect compared to patients who do not experience chronic pain (66). The current study contributes to the larger literature by identifying a possible mechanism linking childhood trauma and pain among individuals with AUD.

Comorbidity of affective disorders and chronic pain has been clearly documented. Increased experiences of pain are related to increases in negative affect and reductions in positive affect (78, 79). Among adults with an AUD, pain and negative affect have also been shown to be closely related. Witkiewitz et al. (51) noticed that in an AUD sample, a higher level of pain was associated with a higher level of negative affect. Kopera et al. (52) demonstrated a mediating effect of poor emotion regulation in the association between negative affect and the experience of severe pain within an AUD sample. Recent research (80) strongly supports physical pain as a predictor in the development of AUD symptoms, as well as a predictor of poor AUD treatment outcomes. Importantly, alcohol may be used both as an analgesic agent to relieve somatic pain, as well as a means to reduce negative affect that commonly accompanies chronic physical pain.

Physical or emotional abuse experienced early in life may disrupt the development of functional emotional regulation strategies. Recent studies provide compelling evidence supporting a higher prevalence of childhood trauma history among various presenting problems: mood and anxiety disorders, self-injurious behavior, suicide attempts, and substance use disorders (26, 81, 82). The impact of child adversity on general well-being in later life may also operate *via* increased risk of somatic diseases (83), as well as altered perception of acute or chronic pain (84). Mechanisms underlying these associations have been linked to alterations within the neuroendocrine system caused by early trauma. Childhood maltreatment (due to prolonged stress) is associated with dysregulation in the hypothalamic–pituitary–adrenal (HPA) axis, which plays a major role in immune system functioning. Disruption in immune system markers [pro- and anti-inflammatory substances (e.g., C-reactive protein, interleukin-6, adiponectin, or cell-mediated immunity)] contributes to increased risk of infections, as well as the development of autoimmune disorders or other chronic disorders, including those associated with pain (85).

To the best of our knowledge, this study is one of the first examinations demonstrating comprehensive associations between childhood trauma, negative affect, emotional dysregulation, and pain among individuals with AUD. A notable exception is a study demonstrating an association between childhood sexual abuse and increased experiences of intense physical pain in adulthood among individuals with AUD (58). Yet, similar studies have been conducted among other clinical samples. For example, current PTSD symptoms (26) and depressive symptoms (86) were found to fully mediate the association between childhood abuse and pain severity.

The model described in the current study represents a possible cascading effect that links childhood emotional abuse with later risk for AUD given the problematic use of alcohol for self-medication purposes. Namely, the experience of emotional abuse during childhood [which could be associated with alcohol drinking by parent(s)] plausibly leads to negative affectivity (higher levels of anxiety or depression) during childhood, adolescence, and adulthood (as supported in the current study). In turn, a lack of constructive strategies to manage anxiety (and other negative emotions) is a core symptom of emotional dysregulation, which is linked to childhood emotional abuse. Both anxiety and emotion regulation deficits are significant risk factors of problematic alcohol drinking. Subsequently, low pain tolerance contributes to emotional dysregulation and negative affect. Moreover, low pain tolerance often leads to alcohol use for analgesic purposes. The long-term reliance on alcohol use to cope with negative affectivity often exacerbates symptomatology (sleep problems, anxiety, irritability, etc.). This represents a vicious cycle whereby pain, emotional dysregulation, and problematic alcohol use reinforce one another and contribute to the development of AUD.

Each of the points depicted in the mediation model constitutes a possible target for therapeutic AUD interventions. Focusing on training of adaptive cognitive and behavioral strategies for the regulation of emotional states was shown to help prevent substance use disorders among individuals with a history of childhood maltreatment. Findings supporting a link between childhood emotional abuse and emotional dysregulation on pain tolerance among individuals with AUD suggest that pain-focused interventions may have particular utility among those in treatment. There is a growing body of literature focusing on the effect of behavioral therapies on addressing pain (87–89). As previously mentioned, pain perception is affected by one's emotional state and there is evidence supporting a common neuroanatomical explanation for this link (53). For example, a study by Seminowicz et al. (90) found that an 11-week Cognitive–Behavioral Therapy (CBT) program for patients with chronic pain promoted an increase in gray matter volume in brain regions that are also associated with pain and emotions (i.e., dorsolateral pre-frontal cortex and ACC). CBT was also shown to provide notable clinical effects for patients with comorbid chronic pain and substance use disorder. That is, not only did CBT improve social, cognitive, emotional, and physical functioning by reducing pain interference on these aspects of life, CBT also had a positive impact on addiction-related outcomes (e.g., alcohol cravings and consumption) (91). In addition, therapeutic work that addresses the impact of childhood trauma on pain perception among individuals with AUD remains an interesting target for future studies.

In our bivariate analyses, we tested possible associations across multiple forms of childhood trauma. Yet, emotional dysregulation was associated only with childhood emotional abuse. This is consistent with a recent study by Christ et al. (28) indicating that only emotional abuse was associated with depressive symptoms and emotion dysregulation. These results are also consistent with those of previous studies demonstrating that emotional abuse is more strongly related to negative affect than other forms of maltreatment (29, 30). Moreover, Christ et al. (28) found that the association between childhood emotional abuse and depressive symptoms was mediated by emotion dysregulation. Children primarily learn emotion regulation skills by observation. For example, a pattern of maladaptive emotion regulation strategies used by the caregiver, negative emotions directed at the child, as well as dismissive behavior toward the child can all have a negative impact on a child's effective development of emotion regulation (28, 92). A recent study conducted by Crosta et al. (93) demonstrated a higher prevalence of childhood trauma and lower resilience (i.e., difficulties adapting to significant sources of stress) among patients with psoriasis compared to healthy controls. Moreover, a significant association was found between childhood trauma and lower resilience among patients with psoriasis. Given that psoriasis is a chronic inflammatory illness associated with increased pain perception, this study is consistent with findings from the current study. Moreover, findings support the assumption that HPA axis dysregulation and impaired catecholamine and neuropeptide release may contribute to the emergence of both emotion dysregulation and low resilience (93). Difficulties managing challenging emotions in a constructive manner may in turn result in the use of maladaptive behaviors, such as use of psychoactive substances, to cope. Work by Wolff et al. (25) and Dutcher et al. (32) provides support for the mediating role of emotional dysregulation in the association between child maltreatment and substance use disorder in later life. The current study extends this work by including pain resilience in these mediated pathways. The current study examined two components of pain—sensitivity and tolerance, but significant effects were only demonstrated for pain tolerance. A possible explanation for this finding may be that sensitivity and tolerance have different psychopathological correlates among individuals with AUD. Importantly, pain is an interoceptive phenomenon. Recent studies suggest that among individuals with AUD, different facets of interoception (accuracy vs. sensibility) may have different clinical correlates (e.g., sleep problems, anxiety) (94, 95), which align with our results concerning different patterns of findings across various facets of pain (tolerance vs. sensitivity). Additional exploration regarding potential differences between pain tolerance and sensitivity is an important future direction.

## Limitations

This is a cross-sectional study, including only participants from an inpatient treatment program for individuals with AUD. A majority of the sample included men with a severe course of AUD, severe emotional dysregulation, a high prevalence of childhood trauma, and negative consequences of drinking. All measures of emotion regulation, pain resilience, and sensitivity, as well as childhood trauma were based on self-report. It would be interesting for future work to assess associations between childhood trauma and behaviorally measured emotion regulation, as well as other measures of pain perception to see if these findings generalize.

In addition, emotion dysregulation was included as a mediator and pain tolerance as a dependent variable in the current study. This decision was due in part to a clearer clinical manifestation of physical pain in comparison to emotional dysregulation. It is often the case that patients (including those with AUD) are more likely to seek treatment for somatic symptoms, such as pain, than for emotional discomfort (96). However, alternative models (i.e., pain tolerance as a mediator of the association between childhood trauma and emotion regulation) are also plausible and warrant further investigation.

The effect sizes of analyzed bivariate associations were relatively small, which may limit the reliability of the findings. Predictors (childhood emotional abuse, anxiety, emotional dysregulation) and the covariates (sex and age) explained only 16% of the variance of current pain resilience. Thus, there are likely other factors that may have a significant impact on pain perception that were not accounted for in this study. This includes a person's general state of health, insight into one's state of health, one's strategies of pain reduction, and disorders associated with altered pain perception (like polyneuropathy, which is common in individuals with AUD). In general, due to the noted methodological limitations, conclusions should be drawn with caution until findings are further replicated. Yet, these findings may still represent a potentially valuable contribution to the understanding of complex associations between childhood trauma, emotion regulation, and pain among individuals with AUD.

## Conclusions

The current study is the first to our knowledge to provide evidence for (1) a significant indirect negative association between childhood emotional abuse and pain tolerance and (2) a mediating role of anxiety and emotional dysregulation on the association between childhood emotional abuse and pain tolerance among individuals with AUD. Addressing possible negative sequelae of childhood emotional abuse, emotion regulation, and pain tolerance may have particular utility among individuals enrolled in AUD therapeutic treatment programs.

## Data Availability Statement

The raw data supporting the conclusions of this article will be made available by the authors, without undue reservation.

## Ethics Statement

The studies involving human participants were reviewed and approved by Bioethics Committee of Medical University of Warsaw (KB/258/2016). The patients/participants provided their written informed consent to participate in this study.

## Author Contributions

JZ, MK, HS, and AJ contributed to the acquisition of data. PK, MK, AJ, and ET provided analysis and interpretation of data. JZ and AJ managed the literature research and wrote the first draft of the manuscript. MK, PK, and ET revised the manuscript and provided substantial input. All authors contributed to the conception, design of the work and approved the content of the final version of the manuscript.

## Conflict of Interest

The authors declare that the research was conducted in the absence of any commercial or financial relationships that could be construed as a potential conflict of interest.
